# Health-related quality of life profiles and their dimension-specific associated factors among Malaysian stroke survivors: a cross sectional study

**DOI:** 10.1186/s12955-021-01847-0

**Published:** 2021-08-30

**Authors:** Hui Jie Wong, Pei Lin Lua, Sakinah Harith, Khairul Azmi Ibrahim

**Affiliations:** 1 School of Nutrition and Dietetics, Faculty of Health Sciences, Gong Badak Campus, Universiti Sultan Zainal Abidin, Terengganu, Malaysia; 2 Faculty Pharmacy, Tembila Campus, Universiti Sultan Zainal Abidin, 22200 Besut, Terengganu, Malaysia; 3 Department of Medicine, Neurology Unit, Hospital Sultanah Nur Zahirah, Ministry of Health, Terengganu, Malaysia

**Keywords:** Health-related quality of life, Association, Stroke, Malaysia, Hospitals

## Abstract

**Background:**

Apart from maximizing functional abilities and independence after stroke, improving overall health-related quality of life (HRQoL) should also become part of the stroke treatment and rehabilitation process goals. This study aimed to assess the HRQoL profiles and explore the dimension-specific associated factors of HRQoL among stroke survivors.

**Methods:**

This was a cross-sectional study of stroke survivors attending post-stroke care clinics in three public hospitals in the states of Pahang and Terengganu, Malaysia. The HRQoL was assessed by EuroQol-5 dimension-5 levels. Data on socio-demographic, clinical profiles, malnutrition risk, and physical activity level were collected through an interviewer-administered survey. Descriptive analyses for HRQoL profiles and multiple logistic regression analyses for its associated factors were performed. Crude and adjusted odds ratios were reported.

**Results:**

A total of 366 stroke survivors were recruited with a mean age of 59 ± 11 years. The most -commonly reported health problems were mobility (85%), followed by usual activities (82%), pain/discomfort (63%), anxiety/depression (51%) and self-care (41%). The mean of the EQ visual analogue scale and the median of the EQ5D summary index was reported at 60.3 ± 14.2 and 0.67 ± 0.37, respectively. Malnutrition risk (mobility, usual activities, and self-care), wheelchair users (self-care and usual activities), speech impairment (usual activities and pain/discomfort), number of stroke episodes (self-care and pain/discomfort), body mass index, physical activity level and types of strokes (usual activities), age and use of a proxy (anxiety/depression), working and smoking status (mobility), were factors associated with either single or multiple dimensions of HRQoL.

**Conclusion:**

Routine malnutrition screening, tailored program for speech therapy, prevention of recurrent stroke, and physical activity promotion should be addressed and further reinforced in current rehabilitation interventions to improve the HRQoL among stroke survivors in Malaysia.

## Introduction

Stroke has a huge negative impact on its survivors, causing permanent functional impairments leading to dependency in daily activities, depressed mood, and social isolation [1]. Consequently, cumulative evidence has consistently demonstrated a lower health-related quality of life (HRQoL) among stroke survivors than those without stroke [2, 3]. Apart from maximizing functional abilities and independence after stroke, improving HRQoL has been gaining importance as part of the stroke treatment and rehabilitation process [4]. HRQoL provides information on a specific dimension of health status (e.g., physical, functional, cognitive, social, and psychological well-being) and evaluation of treatment effectiveness based on the patient’s own perceptive [5].

Findings on HRQoL profiles among stroke survivors have so far been inconsistent. Some studies reported the greatest problems in the psychological domain [6], while others reported in the physical domain [7, 8]. Estimates of HRQoL also varied between studies since socio-economic and cultural differences may have affected the degree of HRQoL. Ultimately, more research regarding HRQoL in different cultures would be meaningful in the generalization of findings.

Besides, it is also important to identify factors associated with HRQoL to make effective management strategies. Previous studies have reported a complex network of physical and psychological factors associated with HRQoL among stroke survivors. These factors included demographic factors (age, female gender, low education level, and poor socio-economic status) and clinical factors (stroke severity, stroke type, recurrent stroke, smoking, comorbidities, functional status/disability, and depression) [9–11]. Although limited, more studies have recently explored the role of physical activity and nutritional status on HRQoL [2, 12].

To our best knowledge, there is no study in Malaysia which had explored the role of comprehensive factors on socio-demographic, clinical, and lifestyle practices on HRQoL status among stroke survivors [13, 14]. Moreover, it is essential to identify which aspects of patients have been affected mainly by their stroke condition and modify the factors affecting HRQoL in specific dimensions to maximize their health improvement and treatment. This information is hard to discern if using the summary index of HRQoL alone. Therefore, this study aimed to examine the HRQoL profiles and their dimension-specific influencing factors among stroke survivors attending outpatient settings in Malaysia's public hospitals.

## Materials and methods

### Setting

A cross-sectional observational study was undertaken between May to August 2019 at post-stroke clinics in the government hospital of Peninsular Malaysia. Based on the statistics of discharge stroke cases in government hospitals in 2015–2016 [15], three hospitals with the highest annual report in the East Coast area of Peninsular Malaysia were selected as the study sites. These hospitals were Hospital Tengku Ampuan Afzan, Hospital Sultan Haji Ahmad Shah, and Hospital Sultanah Nur Zahirah. The study was conducted in the post-stroke care clinics across different departments, including Neurology and Rehabilitation.

### Inclusion and exclusion criteria

The inclusion criteria were as follows: 18 years of age or older, diagnosed with ischaemic or haemorrhagic stroke, and able to communicate in Bahasa Malaysia. Stroke survivors suffering from a transient ischaemic attack, traumatic intracranial haemorrhage, subdural haemorrhage, severe organ failures and psychiatric illness were excluded. Stroke survivors with cognitive deficits or language deficits were included if there was a proxy of the respondent, who is his/her primary caregiver at home. It is recognised that proxy-respondents tend to over-report (more pessimistic) patient's health problems than patients themselves, which may lead to bias [16]. However, the use of proxy indirectly increased the response rates. It allowed us to examine the HRQoL among patients with cognitive and communication disabilities, which were often excluded in other surveys. All eligible patients attending the clinics were invited to participate in this study. The main research assistant interviewed all patients during the survey period.

### Sample size

Based on single mean formula [17], 95% confidence interval, standard deviation 17 for EQ-visual analogue scale (EQ-VAS) [18], margin error of 2, and attrition rate of 20%, a minimum sample size of 347 was required.

### Independent variables and measurements

Data on sociodemographic, clinical, nutritional status and physical activity level were collected. Sociodemographic data included age, sex, marital situation, employment status, household income, and level of education. Clinical history and medical record were examined to collect information on types of strokes, number of stroke episodes, time since stroke, location of stroke, presence of aphasia, number of comorbidities, and wheelchair users in daily life.

Besides, patients were screened for malnutrition risk based on the Malnutrition Risk Screening Tool- Hospital (MRST-H) [19, 20]. Patients who scored two and above were classified as high malnutrition risk. The screening tool included questions on financial dependency, self-feeding ability, unintentional weight loss, and measurements of mid-upper arm circumference and calf circumference. Measurements of body weight and height were conducted to determine the patient's body mass index (BMI). The patient was categorized as obese if his or her BMI more or equal to 27.5 kg/m^2^ based on cut-off points for the Asian population [21]. Knee height, mid-upper arm circumference and previously validated predictive equations were used to estimate the weight and height of stroke survivors who were unable to stand unassisted [22, 23].

Participants' physical activity level after stroke was assessed by the short Malay version of the International Physical Activity Questionnaire (IPAQ) [24, 25]. IPAQ comprises seven items with four activities domains (vigorous, moderate, walking and sitting). The total metabolic equivalent of task (MET)-minutes per week were calculated. Participants were classified into low (< 600 MET-min/week), moderate (600–2999 MET-min/week), or high physical activity level (≥ 3000 MET-min/week) based on the standard scoring protocol [26].

### Dependent variables and measurements

There is no single accepted definition of HRQoL so far. The complexity and diversity of health domains have been reflected by numerous tools available to measure HRQoL, such as the EuroQoL, Short-Form Health Survey, WHOQoL-Bref, and Stroke Specific Quality of Life Scale (SS-QoL). This study used EuroQoL-5 Dimensions- 5 Levels (EQ-5D-5L) considering its availability of validated Malay Version [27], validity in stroke population [28, 29], and limitations of other instruments as reported by previous local study [30]. Besides, the instrument is cognitively undemanding and needs only a few minutes to complete. It consists of two components: the EQ-5D descriptive system and EQ visual analogue scale (EQ-VAS). The ED-5Q-5L description system contains five dimensions: mobility, self-care, usual activities, pain/discomfort, and anxiety/depression. The participants were asked to rate their health today based on a five-point scale, indicating increasing severity of problems (1 = no problems, 2 = slight problems, 3 = moderate problems, 4 = severe problems and 5 = extreme problems). The participants were also asked to self-rate "their health today" based on a scale of 0–100 (from worst to best score) using the VAS. Additionally, an EQ-5D summary index was derived by applying a formula attaches values (weights) to each of the levels in each dimension, with reference to the Malaysian EQ-5D-5L value set published by Shafie et al. (2018) [31]. The EQ-5D summary index can be ranged from 0 (representing death) to 1 (representing full health), and with negative values (representing states worse than death). Results were presented by predefined age-groups (< 50; 50–59; 60–69 and ≥ 70) and by sex (male and female).

### Statistical analysis

Both descriptive and inferential statistics were used with IBM SPSS version 25.0 for Windows. Frequencies and percentages were determined for categorical variables. Mean, and standard deviation was established for numerical variables. A univariate analysis was conducted to investigate dimension-specific HRQoL in stroke survivors. Each dimension in the EQ-5D was categorized into two groups of outcome variables according to previous studies [2, 32]: (1) having problems (slight, moderate, severe and extreme problems) and (2) no problem at all. Socio-demographic characteristics, clinical profiles, nutritional status and physical activity level (independent variables) were compared using the Chi-square test or Fisher's exact test between those having problems and not having problems. Multiple logistic regression was performed to examine predictors of each dimension by including variables with p-value < 0.25 at the univariable analysis. Besides, variables such as age (continuous), sex, and use of proxy respondents were also included in view of their possible confounding effects. Adjusted odds ratios and 95% confidence interval were reported. The fitness of model for each dimension was checked by classification table, Hosmer–Lemeshow's test, and ROC value. Correlations and multicollinearity between variables were checked. P-values of less than 0.05 was considered significant.

## Results

### Respondent’s characteristics

A total of 448 respondents were screened from three hospitals during the survey; however, 30 respondents were excluded based on exclusion criteria, and 20 refused to participate. Hence 398 respondents were recruited. Additionally, 32 of them had an unknown type of stroke (ischaemic or haemorrhagic); thus, only 366 were included in the statistical analysis. There were no missing data in this survey since face-to-face interview was employed and the researcher checked the completeness of the questionnaire before ending each survey. The respondents' socio-demographic background, clinical profiles, nutritional status and physical activity level data are shown in Table 1. Our stroke survivors were predominantly aged more than 60 years old, male, married, completed secondary education level, not working, and earning a household income of less than Malaysian Ringgit (MYR) 3000/ United States Dollar 719. The majority of them had underlying hypertension and dyslipidaemia, while half of them had diabetes mellitus. Most of the patients were first-time stroke survivors, while 20% were recurrent stroke survivors. Almost three quarters (72%) of the survey was completed by the stroke survivors, while the rest (28%) was completed by a proxy of the respondent.

**Table 1:** Socio-demographic characteristics, clinical profiles, nutrition status and physical activity level of the stroke survivors (n = 366)

Variables	n (%)
**Age (mean ± sd)**	59 ± 11
**Age**	
< 60 years	170 (46)
≥ 60 years	196 (54)
**Sex**	
Female	161 (44)
Male	205 (56)
**Marital status**	
Married	272 (74)
Single	22 (6)
Divorced	12 (3)
Widowed	60 (17)
**Education**	
Primary and less	128 (35)
Secondary	196 (54)
Tertiary	42 (11)
**Working status**	
Employed	42 (12)
Unemployed	324 (88)
**Income**	
< MYR 3000	80 (22)
≥ MYR 3000	286 (78)
**Number of comorbidities**	
0–2	156 (43)
3–4	189 (51)
≥ 5	21 (6)
**Obesity (Body mass index ≥ 27.5 kg/m**^**2**^**)**	
Yes	102 (28)
No	264 (72)
**Smoking status**	
Never smoke	226 (62)
Ex-smoker	115 (31)
Smoking	25 (7)
**Type of stroke**	
Ischaemic	284 (78)
Haemorrhagic	82 (22)
**Time since stroke**	
< 12 months	187 (51)
≥ 12 months	179 (49)
**Number of stroke episodes**	
First	292 (80)
Recurrent	74 (20)
**Location of stroke**	
Left	178 (49)
Right	133 (36)
Unspecified	51 (14)
Bilateral	4 (1)
**Aphasia**	
Yes	120 (33)
No	246 (67)
**Wheelchair user**	
Yes	104 (28)
No	262 (72)
**Malnutrition risk**	
High risk (≥ 2 points)	150 (41)
Low risk (< 2 point)	216 (59)
**Physical activity**	
Low (< 600 min MET-min/week)	239 (65)
Moderate and high (≥ 600 MET-min/week)	127 (35)
**Respondents**	
Patient	265 (72)
Proxy	101 (28)

Comorbidities = hypertension, diabetes mellitus, dyslipidaemia, ischaemic heart disease, atrial fibrillation, chronic kidney diseases, liver diseases, pulmonary diseases, arthritis, gout, Parkinson, and mood disorder; Malnutrition risk was assessed by the Malnutrition Risk Screening Tool- Hospital; Physical activity was assessed by the International Physical Activity Questionnaire

MYR, Malaysian Ringgit; MET, metabolic equivalent of task values; min, minutes; sd, standard deviation

### Descriptive analyses of EQ-5D profiles, EQ-VAS and EQ5D index

Table 2 shows the distribution of the problem status of each dimension in the EQ-5D questionnaire. Almost all stroke survivors (98%) reported having problems in either one or multiple dimensions of HRQoL. Our stroke survivors commonly reported having problems (slight to extreme problems) in the dimension of mobility (85%), followed by usual activities (82%), pain/discomfort (63%), anxiety/depression (51%) and self-care (41%). On the other hand, Table 3 displays the mean values of the VAS and median values of EQ5D index stratified by age group and sex. The mean of EQ-VAS and median of EQ5D index was reported at 60.3 ± 14.2 and 0.67 ± 0.37, respectively. There were no significant differences between males and females in both means of EQ-VAS (p = 0.739) and median of EQ5D-index (p = 0.286). The mean of VAS (64–70) and median of EQ5D-index (0.68–0.70) were highest in the < 50 age group for both female and male patients, and they seemed to reduce gradually with increasing age.

**Table 2:** Severity of problems in each EQ-5D-5L dimension among stroke survivors (n = 366)

Dimension	n (%)
**Mobility**	
(a) no problems	55 (15)
(b) slight problems	71 (19)
(c) moderate problems	148 (41)
(d) severe problems	19 (5)
(e) extreme problems	73 (20)
**Self-care**	
(a) no problems	216 (59)
(b) slight problems	33 (9)
(c) moderate problems	54 (15)
(d) severe problems	23 (6)
(e) extreme problems	40 (11)
**Usual activities**	
(a) no problems	67 (18)
(b) slight problems	43 (12)
(c) moderate problems	112 (31)
(d) severe problems	63 (17)
(e) extreme problems	81 (22)
**Pain/discomfort**	
(a) no problems	135 (37)
(b) slight problems	88 (24)
(c) moderate problems	121 (33)
(d) severe problems	20 (5)
(e) extreme problems	2 (1)
**Anxiety/depression**	
(a) no problems	178 (49)
(b) slight problems	96 (26)
(c) moderate problems	69 (19)
(d) severe problems	20 (5)
(e) extreme problems	3 (1)

**Table 3:** Mean of EQ-Visual Analogue Scale and median of EQ5D-index stratified by age group and sex (n = 366)

Variables	Age group									
	Female					Male				
	Total	< 50 (n = 24)	50–59 (n = 49)	60–69 (n = 60)	≥ 70 (n = 28)	Total	< 50 (n = 41)	50–59 (n = 56)	60–69 (n = 70)	≥ 70 (n = 38)
VAS (mean ± sd)	60 ± 14	70 ± 14	59 ± 14	58 ± 13	57 ± 13	61 ± 14	64 ± 15	62 ± 13	58 ± 13	60 ± 16
EQ5D index (median ± IQR)	0.65 ± 0.37	0.70 ± 0.25	0.67 ± 0.19	0.67 ± 0.48	0.43 ± 0.49	0.68 ± 0.40	0.68 ± 0.32	0.67 ± 0.40	0.72 ± 0.40	0.63 ± 0.66

### Associations between socio-demographic, clinical profiles, nutritional status, and physical activity level with specific dimension of HRQoL at univariate analysis

Table 4 shows the associations between socio-demographic, clinical profiles, nutrition status, and physical activity level with a specific dimension of HRQoL at univariate analysis. Factors found to be significantly associated with the physical dimensions of HRQoL (mobility, usual activities, and self-care) were working status, household income, number of stroke episodes, obesity, smoking status, aphasia, wheelchair user, malnutrition risk, physical activity level and use of a proxy respondent. Conversely, younger age, malnutrition risk, use of a proxy respondent and wheelchair user were significantly associated with the psychological dimensions of HRQoL (pain/discomfort and anxiety/depression). Sex, marital status, education levels, number of comorbidities, types of strokes, and time since stroke were not significantly associated with any dimension of HRQoL at univariate analysis.

**Table 4:** Associations between independent variables with specific dimension of HRQoL at univariate analysis (n = 366)

Variables	Mobility		Self-care		Usual activities		Pain/discomfort		Anxiety/depression	
	With problems	No problems	With problems	No problems	With problems	No problems	With problems	No problems	With problems	No problems
	(n = 311)	(n = 55)	(n = 150)	(n = 216)	(n = 299)	(n = 67)	(n = 231)	(n = 135)	(n = 188)	(n = 178)
**Age**										
< 60 years	141 (83)	29 (17)	62 (37)	108 (64)	143 (84)	27 (16)	114 (67)	56 (33)	99 (58)	71 (42)
≥ 60 years	170 (87)	26 (13)	88 (45)	108 (55)	156 (80)	40 (20)	117 (60)	79 (40)	89 (45)	107 (55)
*P*-value	0.311		0.102		0.264		0.145		0.014	
**Sex**										
Female	143 (89)	18 (11)	68 (42)	93 (58)	129 (80)	32 (20)	104 (65)	57 (35)	88 (55)	73 (45)
Male	168 (82)	37 (18)	82 (40)	123 (60)	170 (83)	35 (17)	127 (62)	78 (38)	100 (49)	105 (51)
*P*-value	0.068		0.666		0.491		0.603		0.264	
**Marital status**										
Married	231 (85)	41 (15)	106 (39)	166 (61)	223 (82)	49 (18)	170 (63)	102 (37)	136 (50)	136 (50)
Single	18 (82)	4 (18)	11 (50)	11 (50)	17 (77)	5 (23)	15 (68)	7 (32)	14 (64)	8 (36)
Divorced	10 (83)	2 (17)	6 (50)	6 (50)	11 (92)	1 (8)	11 (92)	1 (8)	10 (83)	2 (17)
Widowed	52 (87)	8 (13)	27 (45)	33 (55)	48 (80)	12 (20)	35 (58)	25 (42)	28 (47)	32 (53)
*P*-value	0.954		0.573		0.749		0.166		0.073	
**Education**										
Tertiary	34 (81)	8 (19)	15 (36)	27 (64)	32 (76)	10 (24)	25 (60)	17 (40)	22 (52)	20 (48)
Secondary	171 (87)	25 (13)	76 (39)	120 (61)	165 (84)	31 (16)	132 (67)	64 (33)	99 (51)	97 (49)
Primary	106 (83)	22 (17)	59 (46)	69 (54)	102 (80)	26 (20)	74 (58)	54 (42)	67 (52)	61 (48)
*P*-value	0.408		0.323		0.366		0.193		0.94	
**Working status**										
Employed	27 (64)	15 (36)	6 (14)	36 (86)	25 (60)	17 (41)	32 (76)	10 (24)	19 (45)	23 (55)
Unemployed	284 (88)	40 (12)	144 (44)	180 (56)	274 (85)	50 (15)	199 (61)	125 (39)	169 (52)	155 (48)
*P*-value	< 0.001		< 0.001		< 0.001		0.062		0.398	
**Income**										
< MYR 3000	67 (84)	13 (16)	25 (31)	55 (69)	61 (76)	19 (24)	52 (65)	28 (35)	38 (48)	42 (52)
≥ MYR 3000	244 (85)	42 (15)	125 (44)	161 (56)	238 (83)	48 (17)	179 (63)	107 (37)	150 (52)	136 (48)
*P*-value	0.729		0.045		0.154		0.693		0.434	
**Number of comorbidities**										
0–2	133 (85)	23 (15)	61 (39)	95 (61)	127 (81)	29 (19)	96 (62)	60 (38)	81 (52)	75 (48)
3–4	162 (86)	27 (14)	79 (42)	110 (58)	156 (83)	33 (17)	120 (64)	69 (36)	94 (50)	95 (50)
≥ 5	16 (76)	5 (24)	10 (48)	11 (52)	16 (76)	5 (24)	15 (71)	6 (29)	13 (62)	8 (38)
*P*-value	0.507		0.718		0.769		0.67		0.562	
**Obesity**										
No	233 (88)	31 (12)	122 (46)	142 (54)	228 (86)	36 (14)	168 (64)	96 (36)	142 (54)	122 (46)
Yes	78 (77)	24 (23)	28 (28)	74 (72)	71 (70)	31 (30)	63 (62)	39 (38)	46 (45)	56 (55)
*P*-value	0.005		0.001		< 0.001		0.739		0.136	
**Smoking status**										
Never smoke	194 (86)	32 (14)	93 (41)	133 (59)	182 (81)	44(19)	149 (66)	77 (34)	120 (53)	106 (47)
Ex-smoker	101 (88)	14 (12)	51 (44)	64 (56)	98 (85)	17 (15)	68 (59)	47 (41)	56 (49)	59 (51)
Smoking	16 (64)	9 (36)	6 (24)	19 (76)	19 (76)	6 (24)	14 (56)	11 (44)	12 (48)	13 (52)
*P*-value	0.009		0.172		0.427		0.351		0.7	
**Types of strokes**										
Ischaemic	237 (83)	47 (17)	112 (39)	172 (61)	226 (80)	58 (20)	181 (64)	103 (36)	141 (50)	143 (50)
Haemorrhagic	74 (90)	8 (10)	38 (46)	44 (54)	73 (89)	9 (11)	50 (61)	32 (39)	47 (57)	35 (43)
*P*-value	0.129		0.263		0.051		0.649		0.221	
**Time since stroke**										
< 12 months	155 (83)	32 (17)	68 (36)	119 (64)	151 (81)	36 (19)	119 (64)	68 (36)	98 (52)	89 (48)
≥ 12 months	156 (87)	23 (13)	82 (46)	97 (54)	148 (83)	31 (17)	112 (63)	67 (37)	90 (50)	89 (50)
*P*-value	0.254		0.066		0.633		0.833		0.683	
**Number of stroke episodes**										
First	244 (84)	48 (16)	108 (37)	184 (63)	235 (81)	57 (19)	178 (61)	114 (39)	145 (50)	147 (50)
Recurrent	67 (91)	7 (9)	42 (57)	32 (43)	64 (87)	10 (13)	53 (72)	21 (28)	43 (58)	31 (42)
*P*-value	0.133		0.002		0.233		0.09		0.194	
**Location of stroke**										
Left	153 (86)	25 (14)	71 (40)	107 (60)	146 (82)	32 (18)	113 (64)	65 (36)	90 (51)	88 (49)
Right	111 (84)	22 (16)	57 (43)	76 (57)	111 (84)	22 (16)	90 (68)	43 (32)	73(55)	60 (45)
Unspecified	44 (86)	7 (14)	21 (41)	30 (59)	40 (78)	11 (22)	26 (51)	25 (49)	22 (43)	29 (57)
Bilateral	3 (75)	1 (25)	1 (25)	3 (75)	2 (50)	2 (50)	2 (50)	2 (50)	3 (75)	1 (25)
*P*-value	0.722		0.899		0.31		0.166		0.406	
**Aphasia**										
No	205 (83)	41 (17)	82 (33)	164 (67)	185 (75)	61 (25)	163 (66)	83 (34)	120 (49)	126 (51)
Yes	106 (88)	14 (12)	68 (57)	52 (43)	114 (95)	6 (5)	68 (57)	52 (43)	68 (57)	52 (43)
*P*-value	0.209		< 0.001		< 0.001		0.074		0.156	
**Malnutrition risk**										
Low	171 (79)	45 (21)	64 (30)	152 (70)	161 (75)	55 (25)	137 (63)	79 (37)	99 (46)	117 (54)
High	140 (93)	10 (7)	86 (57)	64 (43)	138 (92)	12 (8)	94 (63)	56 (37)	89 (59)	61 (41)
*P*-value	< 0.001		< 0.001		< 0.001		0.882		0.011	
**Physical activity**										
Moderate to high	99 (78)	28 (22)	21 (17)	106 (83)	87 (69)	40 (31)	88 (69)	39 (31)	60 (47)	67 (53)
Low	212 (89)	27 (11)	129 (54)	110 (46)	212 (89)	27 (11)	143 (60)	96 (40)	128 (54)	111 (46)
*P*-value	0.006		< 0.001		< 0.001		0.074		0.25	
**Respondents**										
Patient	221 (83)	44 (17)	80 (30)	185 (70)	202 (76)	63 (24)	173 (65)	92 (35)	120 (45)	145 (55)
Proxy	90 (89)	11 (11)	70 (69)	31 (31)	97 (96)	4 (4)	58 (57)	43 (43)	68 (67)	33 (33)
*P*-value	0.172		< 0.001		< 0.001		0.164		< 0.001	
**Wheelchair user**										
No	207 (79)	55 (21)	55 (21)	207 (79)	196 (75)	66 (25)	166 (63)	96 (37)	122 (47)	140 (53)
Yes	104 (100)	0 (0)	95 (91)	9 (9)	103 (99)	1 (1)	65(63)	39 (37)	66 (64)	38 (36)
*P*-value	< 0.001		< 0.001		< 0.001		0.878		0.004	

High malnutrition risk = MRST-H scores ≥ 2 points; Low malnutrition risk = MRST-H scores < 2 points; Moderate to high physical activity level = IPAQ scores ≥ 600 MET-min/week; Low physical activity level = IPAQ scores < 600 MET-min/week; Obesity = Body mass index ≥ 27.5 kg/m^2^**;** Comorbidities = hypertension, diabetes mellitus, dyslipidaemia, ischaemic heart disease, atrial fibrillation, chronic kidney diseases, liver diseases, pulmonary diseases, arthritis, gout, Parkinson, and mood disorder; Chi-square test or Fisher's exact test was applied with significance level set at p < 0.05

MET, metabolic equivalent of task values; MRST-H, Malnutrition Risk Screening Tool-Hospital; IPAQ, International Physical Activity Questionnaire

### Dimension-specific associated factors of EQ-5D-5L

Table 5 shows the dimension-specific associated factors of EQ-5D-5L using multivariable logistic regression after adjusted for confounding variables. This study revealed that high malnutrition risk patients were two to three times more likely to report having problems in all physical dimensions of HRQoL namely mobility, self-care and usual activities. Similarly, wheelchair-bound stroke survivors were 14 to 34 times higher risk of having problems in self-care and usual activities dimensions than their counterparts. Besides, stroke survivors with recurrent stroke were twice more likely to report problems in self-care and pain/discomfort. Stroke survivors with aphasia were at higher odds of reporting problems in the usual activities yet lower odds of reporting problems in pain/discomfort. Haemorrhagic stroke and low physical activity levels were significantly associated with higher odds of having problems in usual activities. Unemployment and use of a proxy respondent were found to be associated with higher odds of having problems in mobility and anxiety/depression dimension, respectively. Conversely, older age, obesity, and active smoking was associated with lower odds of having problems in anxiety/depression, usual activities, and mobility dimension, respectively.

**Table 5:** Dimension-specific influencing factors of eq-5d among stroke survivors (n = 366) using multivariable logistic regression analysis

	Mobility aOR (CI)	Self-care aOR (CI)	Usual activities aOR (CI)	Pain/discomfort aOR (CI)	Anxiety/Depression aOR (CI)
**Age**	**–**	**–**	**–**	**–**	0.97 (0.95, 0.99)*
**Working status**					
Employed (ref)	1	–	–	–	–
Unemployed	2.96 (1.40, 6.26)#				
**Types of strokes**					
Ischaemic (ref)	–	–	1	–	–
Haemorrhagic			2.32 (1.01, 5.31)*		
**Number of stroke episodes**					
First (ref)	–	1	–	1	–
Recurrent		2.09 (1.07, 4.08)*		1.86 (1.04, 3.31)*	
**Malnutrition risk**					
Low risk (< 2 points)(ref)	1	1	1	–	–
High risk (≥ 2 points)	3.07 (1.46, 6.46)#	1.78 (1.01, 3.14)*	2.46 (1.19, 5.10)*		
**Aphasia**					
No (ref)	–	–	1	1	–
Yes			4.35 (1.76, 10.77)#	0.59 (0.37, 0.94)*	
**Smoking status**					
Never smoke (ref)	1	–	–	–	–
Ex-smoker	1.15 (0.58, 2.32)				
Smoking	0.29 (0.11, 0.76)*				
**Obesity (BMI ≥ 27.5 kg/m**^**2**^**)**					
No (ref)	–	–	1	–	–
Yes			0.53 (0.29, 0.99)*		
**Physical activity level**					
Moderate to high (ref)	–	–	1	–	–
Low			2.12 (1.15, 3.90)*		
**Wheelchair user**					
No (ref)	–	1	1	–	–
Yes		33.85 (15.87, 72.22)**	13.55 (1.77, 103.84)*		
**Respondents**					
Patient (ref)	–	–	–	–	1
Proxy					2.60 (1.60, 4.23)**
Hosmer–Lemeshow’s test	0.961	0.811	0.982	1	0.691
Classification Table (%)	86.1	82.5	83.3	63.1	59.3
ROC	0.688	0.832	0.812	0.574	0.64

High malnutrition risk = MRST-H scores ≥ 2 points; Low malnutrition risk = MRST-H scores < 2 points; Moderate to high physical activity level = IPAQ scores ≥ 600 MET-min/week; Low physical activity level = IPAQ scores < 600 MET-min/week; Multivariate logistic regression is applied. Adjusted for variables found to have p < 0.25 at univariate analysis in Table 4, age (continuous), sex, and use of a proxy respondent

BMI, body mass index; MET, metabolic equivalent of task values; min, minutes; MRST-H, Malnutrition Risk Screening Tool-Hospital; IPAQ, International Physical Activity Questionnaire; aOR, adjusted odd ratio; CI, confidence interval

*p < 0.05

^#^p < 0.005

**p < 0.001

## Discussion

This study showed that stroke survivors reported having problems in different dimensions of HRQoL, with the highest frequency reported in the mobility dimension and the lowest in the self-care dimension. High malnutrition risk, wheelchair users, presence of aphasia, recurrent stroke, low physical activity level, haemorrhagic stroke, and unemployment were associated with higher odds of having problems in the physical dimensions of HRQoL (mobility, usual activities, and self-care). Conversely, obesity and active smoking were associated with lower odds of having problems in the physical dimensions of HRQoL. The presence of aphasia, recurrent stroke, younger age, and proxy use was significantly associated with the psychological dimensions of the HRQoL (pain/discomfort and anxiety/depression).

The overall HRQoL as reported using VAS and summary of EQ5D-index was 60 and 0.67, respectively. Although there is no specific classification of VAS according to the EQ-5D manual, the reported value of 60 indicating average or moderate health status. This finding was consistent with previous studies in Korea (61) [33], yet lower than that reported in Sweden and Malaysia (70–71) [18, 34], and higher than that reported in Spain (50) [35]. Additionally, the summary of EQ5D-index (0.67) obtained in this study was in line with findings in France (0.63) [36] and US (0.69) [37], but lower than those in Norway (0.74) [9] and Singapore (0.83) [38].

In general, more than half of our stroke survivors reported having problems in almost all dimensions of HRQoL, indicating poor health status from the patient's perspective. The sequence of the most reported health problem by stroke survivors in this study (mobility, usual activities, pain/discomfort, anxiety/depression, and self-care) was not so different from findings from a previous study in Australia (usual activities 58%, mobility 57%, pain/discomfort 49%, anxiety/depression 47%, and self-care 33%) [7]. Of the five domains, mobility and usual activities domains were the most reported problems. The high prevalence is probably because most of the respondents were recruited from the rehabilitation centre, where many suffered from specific limb weaknesses that require rehabilitation services. Almost 30 percent of the stroke survivors were wheelchair users, while others complained of gait, balance and fatigue issues while walking. Walking impairment often have a negative impact on activities of daily living and social participation [39]. The usual activity domain includes working, housework, family or leisure activities, which requires an ability to move around. Besides, a previous local study showed that almost one-fifth of the stroke survivors discharged from rehabilitation clinics of the hospital demonstrated unfavourable scores for mobility and activities of daily living (ADL) [14].

In this study, high malnutrition risk was significantly associated with higher odds of having problems in all physical dimensions of HRQoL. This is in line with a prospective study in Germany, which found a significant negative association between malnutrition risk and EQ-5D index at one year after stroke survivors ward discharge [12]. Studies on the relationship between malnutrition risk and HRQoL among stroke population were relatively limited [12, 40], although evidence exists to indicate higher malnutrition risk and lower HRQoL among the elderly population [41, 42]. However, it remains unclear whether malnutrition was the cause or the consequence or if the association was through mediators [43]. Malnutrition contributed to the development of sarcopenia with loss of lean body mass and muscle function [44]. Consequently, it decreases the physical functioning of stroke survivors, especially in mobility, self-care and usual activities. Second, negative emotions and depression might affect food intake [45], which might interact with the nutritional status [43]. Given the high prevalence of malnutrition risk and its significance with HRQoL, it is important to monitor the stroke survivor's nutritional status routinely even after discharge from the ward. Prompt and optimal nutritional strategies should be taken to prevent further deterioration of nutritional status among these high malnutrition risk groups.

On the other hand, stroke survivors with aphasia or language deficits were at higher risk of having problems in the self-care dimension, yet at lower risk of having pain/discomfort problem. This is similar to the finding by other studies that reported a significant negative impact of aphasia on HRQoL among stroke survivors [3, 46]. Stroke survivors with aphasia faced significant difficulties in participating in complex social activities such as work, family and leisure activities that involve other people [46]. Moreover, the presence of speech and language difficulties may hinder effective communication about self-care needs, food preferences and expression of pain and discomfort [47]. As a result, stroke survivors with aphasia may not obtain the appropriate care and treatment they need from the caregiver or healthcare members.

Interestingly, this study found that obese stroke survivors were less likely to report problems in the usual activities dimension. The relationship between BMI and HRQoL have been inconclusive, with some studies reporting no significant associations [12, 33]. Conversely, recent observational studies have suggested that overweight or obese patients had better survival rates, functional outcomes, and less stroke recurrence than those normal or underweight [48, 49]. However, these studies were subjected to several methodological limitations due to their observational study design, selection bias, survival bias, BMI limitation in indicating obesity, and inaccurate body weight measurement [49]. The relationship between obesity and better functional status should be interpreted in caution as it may be subjected to collider bias or driven by malnutrition among some individuals in the non-obese group. Moreover, body composition changes were more prominent in stroke survivors than in healthy individuals [50]. Significantly lower bone mineral content and lean mass was found in the stroke survivors (but was unlikely to be captured by BMI alone) as compared with their counterparts of similar age, regardless of the presence of limb paralysis [50]. This warrants future prospective studies to explore the impact of changes in BMI, body composition and malnutrition risk on stroke survivors’ functional outcomes.

Low physical activity level was significantly associated with all three physical dimensions of HRQoL, which are aligned with results from a previous study in Korea [2]. However, its effect remained significant only in the usual activities dimension after adjusting for other variables. The causal relationship between physical activity and HRQoL is not known. Poor HRQoL probably leads to low physical activity and the relationship may be confounded by stroke severity. Conversely, increased physical activity was associated with better post-stroke HRQoL [51, 52]. The benefits of physical activity on physical and mental health have been shown, such as improved motor ability and fitness levels, improved emotional wellbeing and reduced depression level [53]. Therefore, more efforts are needed to increase further patient's participation in physical activity and exercise along with the conventional rehabilitation treatment.

Furthermore, stroke survivors who ambulate using wheelchair were more likely to have problems in the physical dimensions of HRQoL such as self-care and usual activities. Similarly, those who suffered from recurrent stroke were more likely to have pain/discomfort and self-care problems. Recurrent stroke is common among stroke survivors (20%), and it is often more severe and disabling than the first episode [54]. We observed that 40% of the recurrent stroke survivors were ambulating using a wheelchair, and 16% of them required tube feeding and were thus more likely to have self-care problems. Additionally, pain and discomfort observed among recurrent stroke cases was probably associated with shoulder pain, central post-stroke pain, pain from spasticity or muscle stiffness, and discomfort from tube feeding [55]. Therefore, apart from optimizing the stroke rehabilitation process, future stroke prevention strategies must be implemented to enhance HRQoL among stroke survivors.

On the other hand, smoking was associated with lower odds of having problems in the mobility dimension. In other words, persistent smoking was more commonly observed in those without mobility issues or who have better functional status as observed by other studies [56, 57]. Financial constraints, mobility impairment and lack of social gathering might have restricted patients’ access to cigarettes supply since many were dependent on others for income sources or were having walking impairment during the survey. Persistent smoking in stroke survivors was undesirable since smoking has been associated with increased risk of future cardiovascular and death events as well as poorer functional outcomes [58, 59]. Thus, more intensive strategies should be implemented to assist in smoking cessation among persistent smokers.

Other significant factors associated with HRQoL were non-modifiable factors such as age (anxiety/depression), unemployment (mobility), haemorrhagic stroke (usual activities), and use of proxy (anxiety/depression), with each affecting different dimension of HRQoL respectively. We found that anxiety/depression was more commonly found in younger stroke survivors. The mean age of stroke onset in this study was 57, which was 15 years younger than those in developed countries (72 years old) [60]. Moreover, 18% of the stroke survivors were young stroke with stroke onset at less than 50 years old. Stroke is a disastrous disease, and it can bring a sudden change in life events and a decline in health status both physically and psychologically. It is also associated with heavier financial burden through loss of patient’s job, cost of nursing care and medical bills [61], especially for those still in the working age. Consequently, it may induce stress and anxiety among these young stroke survivors.

This study also showed that stroke survivors who were not working during the survey were more likely to have mobility issues. Among these stroke survivors who were not working, 23% of them were unemployed (< 60 years), 42% were retirees, and 27% were housewives. Ability to walk with good balance and walking speed greatly affected stroke survivor’s ability to return to work, particularly among the young stroke survivors [62, 63]. Additionally, we also observed that some stroke survivors had early retirement due to significant physical impairments.

The use of a proxy was associated with higher odds of having problem in the anxiety/depression dimension as compared to that of self-reporter. A considerable proportion of stroke survivors cannot self-report because of cognitive, communication, and disability problems. Dorman et al. (1997) have shown moderate agreement between responses from patients and those from their proxies for the physical domains, but less good for the more subjective domains [64]. In fact, some studies demonstrated that proxy bias is most significant in the psychological dimension of HRQoL. Thus, proxy responses may not be suitable for the anxiety dimension [65, 66]. Therefore, results in the measurement of psychological dimension of HRQoL need to be interpreted with caution when there is a considerable amount of proxy respondents in a survey.

Notably, this study did not find any association between sex and HRQoL. The influence of sex on the HRQoL of stroke survivors have remained inconsistent, with some studies reported lower EQ-5D scores among females [11, 67], while others showed no associations [38, 68].

### Limitations

This study has several limitations. First, this finding's generalisability is limited because they were based on an observational cross-sectional study design. Second, variables on the severity of stroke (as indicated by the National Institutes of Health Stroke Scale*)* and degree of disability (as indicated by the Modified Rankin Scale) were not examined in this study. Variables on NIHSS and MRS were either largely missing or required a manual search in the patient’s record, which requires more time and human resources. Despite these limitations, this is probably among the first few multi-centred studies investigating the association between widely varied variables (socio-demographics, clinical profiles, nutritional status, and physical activity) with each dimension of HRQoL among stroke survivors using the EQ-5D-5L instrument. Therefore, we believe that it may shed some light on the causes of poor HRQoL after stroke.

## Conclusion

In summary, high malnutrition risk, aphasia, low physical activity, wheelchair user, obesity, younger age, unemployment, recurrent stroke, haemorrhagic stroke, smoking, and use of a proxy respondent were significantly associated with different dimensions HRQoL among stroke survivors. Routine malnutrition screening, tailored program for speech therapy, prevention of recurrent stroke, and physical activity promotion should be addressed and further reinforced in current rehabilitation interventions to improve the HRQoL among stroke survivors in Malaysia.

## Data Availability

The data that support the findings of this study are available from the corresponding author, but restrictions apply to the availability of these data and are not publicly available.

## Abbreviations

BMI: Body mass index
EQ-5D-5L: EuroQoL-5 Dimensions- 5 Levels
EQ-VAS: EQ-visual analogue scale
HRQoL: Health-related quality of life
IPAQ: International Physical Activity Questionnaire
MET: Metabolic equivalent of task
MRST-H: Malnutrition Risk Screening Tool-Hospital
MYR: Malaysian Ringgit
ROC: Receiver operating characteristic
